# Modulating PD-L1 expression in multiple myeloma: an alternative strategy to target the PD-1/PD-L1 pathway

**DOI:** 10.1186/s13045-018-0589-1

**Published:** 2018-03-27

**Authors:** Rosemarie Tremblay-LeMay, Nasrin Rastgoo, Hong Chang

**Affiliations:** 1 Laboratory Hematology/Laboratory Medicine Program, University Health Network, University of Toronto, Toronto, Canada; 2 Division of Molecular and Cellular Biology, Toronto General Research Institute, Toronto, Canada; 3 Department of Talent Highland, First Affiliated Hospital of Xi’an Jiao Tong University, Xian, China; 4 Laboratory Hematology, Toronto General Hospital, 200 Elizabeth Street, 11th floor, Toronto, ON M5G 2C4 Canada

**Keywords:** Immune checkpoint inhibitors, PD-L1, PD-1, Multiple myeloma, Histone deacetylase, Bromodomain and extraterminal inhibitors, Oncolytic reovirus, MicroRNA

## Abstract

Even with recent advances in therapy regimen, multiple myeloma patients commonly develop drug resistance and relapse. The relevance of targeting the PD-1/PD-L1 axis has been demonstrated in pre-clinical models. Monotherapy with PD-1 inhibitors produced disappointing results, but combinations with other drugs used in the treatment of multiple myeloma seemed promising, and clinical trials are ongoing. However, there have recently been concerns about the safety of PD-1 and PD-L1 inhibitors combined with immunomodulators in the treatment of multiple myeloma, and several trials have been suspended. There is therefore a need for alternative combinations of drugs or different approaches to target this pathway. Protein expression of PD-L1 on cancer cells, including in multiple myeloma, has been associated with intrinsic aggressive features independent of immune evasion mechanisms, thereby providing a rationale for the adoption of new strategies directly targeting PD-L1 protein expression. Drugs modulating the transcriptional and post-transcriptional regulation of PD-L1 could represent new therapeutic strategies for the treatment of multiple myeloma, help potentiate the action of other drugs or be combined to PD-1/PD-L1 inhibitors in order to avoid the potentially problematic combination with immunomodulators. This review will focus on the pathophysiology of PD-L1 expression in multiple myeloma and drugs that have been shown to modulate this expression.

## Background

Multiple myeloma (MM) is the second most common hematological malignancy [1]. Even with recent advances in therapy regimen, patients commonly develop drug resistance and relapse [2]. Patients that become refractory to conventional therapies have a poor outcome [3]. There are ongoing efforts in finding new therapeutic strategies as well as predictive biomarkers for drug resistance and outcome in MM.

Programmed death-ligand 1 (PD-L1), also known as B7-H1 and CD274, is a cell-surface glycoprotein that links to receptor programmed cell death-1 (PD-1) on T lymphocytes. It is normally involved in peripheral tolerance [4], as well as termination of immune response and immune exhaustion, which occurs when cells are exposed to a prolonged antigen stimulus [5–7]. It is constitutively expressed at low levels on antigen-presenting cells, vascular endothelial cells, pancreatic islet cells, as well as in sites of immune privilege (placenta, testes, eye) [7]. It is also expressed in a large number of malignancies [5, 8]. Expression of PD-L2, the other ligand of PD-1, is more restricted and is found on dendritic cells and macrophages after activation [7].

The relevance of targeting the PD-1/PD-L1 axis in MM has been demonstrated in pre-clinical models, and many clinical trials are ongoing. There are two major PD-1 inhibitors being studied in MM: nivolumab and pembrolizumab. There is one major PD-L1 inhibitor currently studied in MM: durvalumab. Table 1 summarizes the main characteristics of these drugs.

**Table 1:** Pharmacological characteristics of the main PD-1 and PD-L1 inhibitors studied in MM

Generic name (Manufacturer)	Ig class	Terminal half-life	Target epitope	Main toxicities	Ref.
PD-1 inhibitors					
Nivolumab *(Bristol-Myers Squibb)*	IgG4	26.7 days	N-loop of PD-1	Immune-mediated endocrinopathies, gastrointestinal, hepatic, pulmonary, renal, skin adverse reactions; immune-mediated encephalitis; infusion reactions; complications in patients receiving allogenic hematopoietic stem cell transplantation after exposure to nivolumab	[129, 130]
Pembrolizumab (*Merck*)	IgG4 kappa	26 days	C’D loop of PD-1	Immune-mediated pneumonitis, colitis, hepatitis, nephritis and renal dysfunction, endocrinopathies, skin reactions; infusion-related reactions	[131–133]
PD-L1 inhibitors					
Durvalumab (*AstraZeneca*)	IgG1	17 days	Mainly front-β-sheet face constituted by A, G, and F strands of the IgV domain of PD-L1	Immune-mediated pneumonitis, hepatitis, colitis, endocrinopathies, nephritis, rash; infections; infusion related reactions	[134, 135]

There have recently been concerns about the safety of PD-1 inhibitors in combination treatments with immunomodulators for MM. Based on these concerns, several clinical trials involving PD-1 and PD-L1 inhibitors combined with pomalidomide and lenalidomide have been suspended or put on clinical hold pending further results. Trials evaluating combination of PD-1/PD-L1 inhibitors with other classes of drugs are still ongoing. There is therefore a justification for exploring alternative combinations of drugs or different approaches to target this pathway. The rationale for the adoption of new strategies targeting the expression of PD-L1 on MM cells will be reviewed, and new therapeutic avenues will be discussed.

### Expression of PD-L1 in MM

A number of studies have demonstrated that PD-L1 expression is present in plasma cells from patients with MM, but not from healthy donors [9–13], and its expression is higher in patients with MM than monoclonal gammopathy of undetermined significance (MGUS) [11, 14]. A correlation between expression of PD-L1 and increased risk of progression to clinical MM has been reported by Dhodapkar et al. [15]. Nevertheless, there are discordant studies. One study showed variable expression of PD-L1 in clonal plasma cells from MM and MGUS patients; however, patients with persistent minimal residual disease showed high expression of PD-L1 and PD-1 [16]. Another study in 351 MM patients identified very heterogeneous expression of PD-L1 transcript levels, with MM patients as a group showing no significant overexpression compared to normal plasma cells [17].

### Mechanisms of regulation of PD-L1 in MM

**Fig. 1:**
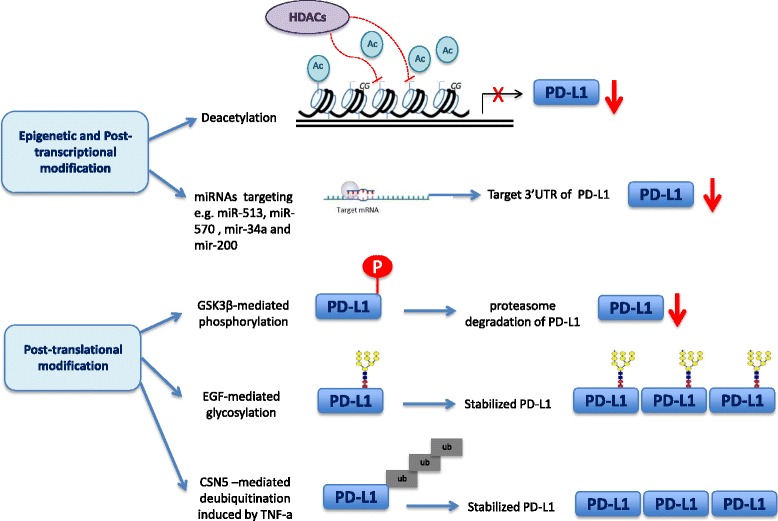
Mechanisms of epigenetic, post-transcriptional, and post-translational modifications of PD-L1. The mechanisms of epigenetic and post-transcriptional modification of PD-L1 include histone deacetylation of the PD-L1 promoter region by HDACs and regulation by microRNAs (miRNAs), such as miR-34a, miR-200, miR-513, and miR-570. Both of these mechanisms result in downregulation of PD-L1. There are several mechanisms of post-translational modification in PD-L1 protein: GSK3β induces phosphorylation-dependent proteasome degradation of PD-L1, leading to downregulation of PD-L1; EGF-mediated glycosylation inactivates GSK3β, which stabilizes PD-L1; CSN5-mediated deubiquitination induced by TNF-α, which leads to stabilization of PD-L1

There is a large body of literature addressing the mechanisms that regulate PD-L1 expression in cancer cells [5, 18–21]. These can be classified as extrinsic, largely associated with pro-inflammatory cytokines, or intrinsic, mediated by various oncogenic and transcriptional pathways such as PTEN, mTOR, or PI3K pathways [21]. While PD-L1 mRNA is broadly found in normal tissues, protein expression is mostly restricted to macrophage-like cells and rare tissues such as the placenta, suggesting heavy post-transcriptional regulation [18, 21, 22]. Figure 1 outlines the mechanisms of epigenetic and post-transcriptional modification, as well as the mechanisms of post-translational modification of PD-L1 that have been described in the literature. Mechanisms of epigenetic and post-transcriptional modification include deacetylation [23, 24] and regulation by microRNAs (miRNAs), such as miRNA-34a [25], miRNA-200 [26], miRNA-513 [27], and miRNA-570 [28]. Both of these mechanisms result in downregulation of PD-L1. There are also some post-translational modifications which regulate the PD-L1 protein level. Glycogen synthase kinase 3β (GSK3β) induces phosphorylation-dependent proteasome degradation of PD-L1, leading to downregulation of PD-L1 [29]. Epidermal growth factor (EGF)-mediated glycosylation inactivates GSK3β [29–31], while TNF-α induces COP9 signalosome 5 (CSN5)-mediated deubiquitination [32], both of which lead to stabilization of PD-L1protein in tumor cells.

Other regulatory pathways of PD-L1 have been shown to be involved in MM. Production of IL-6 by stromal cells is of relevance in MM, as evidenced by the induction of PD-L1 by IL-6 on human myeloma cell lines, which can be downregulated by inhibiting STAT3, MEK1/2, or JAK2 [11]. PD-L1-induced resistance to melphalan and bortezomib via reverse signal of PD-L1 bound to PD-1 was partially due to the activation of PI3K/AKT pathway [33]. The induction of PD-L1 by interferon-γ (IFN-γ) or Toll-like receptor ligands via MyD88/TRAF6 and MEK/ERK/STAT1 pathways has also been demonstrated [14]. A very recent study reported that eosinophils in the tumor microenvironment secrete A proliferation-inducing ligand (APRIL), which promotes MM growth and upregulates PD-L1 and PD-L2 on MM cells [34]. Osteoclasts and myeloid cells in the BM also secrete APRIL, which was found to induce PD-L1 expression in MM cells via its receptor B-cell maturation antigen (BCMA) in a MEK/ERK pathway [35]. These pathways represent potential targets for treatment of MM.

### PD-1/PD-L1 inhibition in MM

**Fig. 2:**
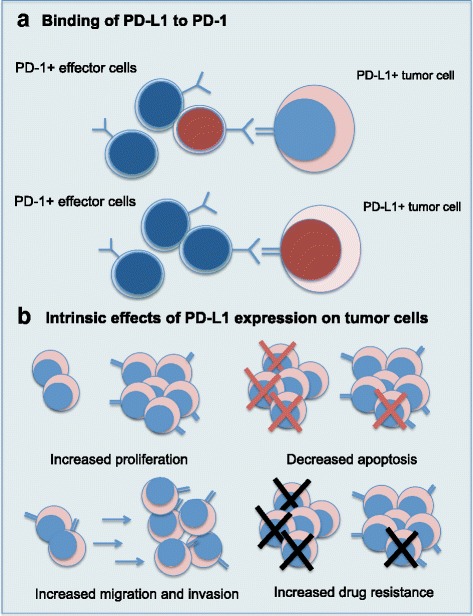
Intrinsic and extrinsic effects of PD-L1 expression on tumor cells. **a** PD-L1 can bind to PD-1 expressed on immune effector cells. This interaction induces T-cell apoptosis, T-cell exhaustion, selective suppression of tumor-specific T-cells (“molecular shield” effect) and regulatory T cells. It can also inhibit the function of NK and invariant NK T cells. The binding of PD-L1 and PD-1 also generates a reverse signal in the tumor cell that has a pro-survival effect and induces resistance to chemotherapy. **b** The expression of PD-L1 on tumor cells is associated with increased proliferation, decreased apoptosis, increased migration and invasion, and increased drug resistance

Studies in cell lines and animal models have provided sufficient evidence for the potential therapeutic effect of PD-1/PD-L1 axis blockade in MM [9, 10, 13, 16]. However, an initial Phase 1b study with nivolumab monotherapy showed no tumor response in relapsed/refractory MM [36]. Similarly, the best response observed in a phase 1B trial of pembrolizumab in monotherapy for RRMM (NCT01953692/KEYNOTE-013) was stable disease in 57% (17/30) of patients [37]. It has been hypothesized by some authors that the efficacy of PD-1 blockade is linked to the mutational burden and infiltrating effector cells in the tumor bed, which happen to be relatively lower in MM compared to solid tumors [38–40]. It has also been suggested that this could be due to the fact that T-cells in MM predominantly exhibit a telomere-independent senescence phenotype or senescence-associated secretory phenotype rather than the exhausted phenotype that can be revived by PD-1/PD-L1 axis blockade [41, 42]. A recent report described high levels of Eomes in the myeloma antigen-specific CD8+ T-cells, indicating profound functional exhaustion that might not be revived by PD-1/PD-L1 blockade alone [43]. Kelly et al. found heterogeneous expression of PD-L1 in MM patients and hypothesized that combination with a drug upregulating PD-L1 in tumor cells could potentiate the effect of PD-1/PD-L1 inhibition [17]. These findings suggest that PD-1/PD-L1 blockade in MM should be part of therapeutic strategies combining multiple drugs, and efforts have been made to identify relevant combinations in MM [44]. Another element to consider is that the effect of PD-L1 on tumor physiology is mediated not only through extrinsic binding to PD-1 on effector cells, but also through intrinsic effects on the tumor cells themselves (Fig. 2). This has potential implications for therapeutic strategies.

### Effects of PD-L1 on tumor physiology

#### Binding of PD-L1 to PD-1

PD-L1 can induce T-cell apoptosis in vitro and in vivo via binding to PD-1 [8]. The binding can also induce T-cell exhaustion, resulting in a deficient cellular immunity [45]; however, these cytotoxic T-cells remain functional and can potentially be reinvigorated with blockade of PD-1/PD-L1 axis [22, 46]. PD-1/PD-L1 interaction also leads to selective suppression of tumor-specific T-cells, since PD-1 is expressed on T-cells upon activation after encountering tumor antigen, thereby forming a “molecular shield” allowing the tumor to evade immune response [7, 22]. PD-L1 can also induce and maintain regulatory T cells, which will promote suppression of anti-tumor immune response [47]. It has also been shown that PD-1 is expressed on natural killer (NK) cells in MM patients and that PD-1/PD-L1 interaction inhibits the response of NK cells to MM cells [9]. The PD-1/PD-L1 axis is also involved in the induction and maintenance of anergy in invariant NK T cells [48, 49]. The principle of PD-1/PD-L1 inhibition is therefore to block this interaction and revive the host immune response against tumor cells. Binding of PD-L1 to PD-1 also induces a reverse signal in MM cells that is pro-survival and induces chemotherapy resistance [33].

#### Intrinsic features associated with PD-L1 expression

##### Increased cell proliferation

Tamura et al. showed that RPMI8226 and KMS-34 MM cells expressing surface PD-L1 had a proliferative advantage over cells from the same line that did not express PD-L1, as demonstrated by higher fraction of cells in the G2/M phase, higher levels of BrdU incorporation, percentage of Ki67 positivity, and more rapid proliferation in cell cultures [11]. Conversely, knockdown of PD-L1 in MOST-1 MM cells was associated with slower proliferation and decreased BrdU incorporation [33].

The effect of PD-L1 overexpression on cell proliferation was also identified in other types of cancer cells, such as colorectal carcinoma cell line, gastric cancer cell lines, pancreatic cell lines, as well as an ovarian carcinoma cell line [50–53]. Overexpression of PD-L1 was associated with increased viability in an esophageal cancer cell line [54]. Moreover, histological assessment of 69 breast cancer samples showed a significant association between PD-L1 expression by immunohistochemistry (IHC) and mitotic index. Double staining for PD-L1 and Ki67 IHC in the majority of PD-L1+ cells also highlighted this relationship [55]. It was also shown in fibroblast cell lines that PD-L1 is upregulated when the cells are cultured in the presence of a strong mitogen (EGF). There was complete abolishment of PD-L1 in quiescent cells, with restoration after cell proliferation was reinstated [55]. Notably, an in vivo study has shown that tumor growth was decreased in a mouse model of gastric cancer with knockdown of PD-L1 [51].

##### Increased levels of anti-apoptotic proteins and decreased apoptosis

In RPMI8226 cells, PD-L1+ cells had markedly higher expression levels of Bcl-2 and FasL expression compared to PD-L1- cells. The levels of intracellular Bcl-2 and cell-surface FasL proteins were also higher in PD-L1+ cells [11]. Conversely, knockdown of PD-L1 in MOST-1 cells downregulated expression of anti-apoptotic genes (BCL2 and MCL1) [33] and silencing of PD-L1 in colorectal cancer cell line and gastric cancer cell lines was associated with an increased apoptotic index [50, 51]. In addition, PD-L1 was shown to inhibit the apoptosis of malignant melanoma initiating cells and could contribute to maintaining the stem cell-like properties of these cells [56]. PD-L1 has also been shown to sustain stem cell-like features in breast cancer cells [57].

##### Increased migration and invasion

Using standard Matrigel-coated or uncoated transwell chamber assays, Shi et al. showed that knockdown of PD-L1 in colorectal cancer cell line, HCT116, was associated with reduced migration and invasion ability, with a reduced invasive index [50]. Similar findings were observed in gastric cancer cell lines as well as an esophageal cancer cell line, where it also promoted an epithelial to mesenchymal transition phenotype [51, 54]. Moreover, increased motility and invasiveness was shown in melanoma cell lines expressing PD-L1 [58]. PD-L1 was also shown to induce tumor formation in malignant melanoma initiating cells [56].

##### Increased drug resistance

Dexamethasone and melphalan induced marked apoptosis in PD-L1- RMPI8226 cells, but not in PD-L1+ cells. Apoptosis was also observed in KMS-28PE cells that did not express PD-L1, but not in PD-L1-transduced cells [11]. Conversely, knockdown of PD-L1 on MOST-1 cells was associated with increased apoptosis when treated with melphalan. Simple blockade of PD-L1 on MOST-1 cells using anti-PD-L1 did not affect apoptosis (nor BrdU incorporation) [33]. Additionally, significant upregulation of PD-L1 was observed in melanoma cell lines with acquired resistance to BRAF or MEK inhibitors [58]. The cytotoxic activity of cytokine-induced killer cells was increased with knockdown of PD-L1 in gastric cancer cell lines [51].

### PD-L1 expression and clinicopathological features

Higher expression of PD-L1 was associated with a higher percentage of infiltration of the bone marrow in MM patients. PD-L1 expression levels were also often upregulated in patients that relapsed or became refractory to therapy [11]. In a prospective study, expression of PD-L1 on tumor and infiltrating T-cells was associated with a higher risk of progression of MGUS to clinical malignancy [15]. Moreover, serum levels of soluble PD-L1 were shown to predict treatment response and progression-free survival in 81 newly diagnosed MM patients [59] and high soluble PD-L1 levels in bone marrow plasma from 61 MM patients were associated with shorter response after autologous stem cell transplant (ASCT) and shorter overall survival [60]. There was no statistically significant difference in high-risk cytogenetic abnormalities between patients with high versus normal-to-low soluble PD-L1 levels. Both high-soluble PD-L1 levels and high-risk cytogenetic abnormalities were independent factors for shorter response period after ASCT and shorter overall survival [60].

Multiple studies have demonstrated the prognostic significance of PD-L1 expression by IHC in solid tumors [61–63] and in some hematological malignancies [64–66]. Immunohistochemistry (IHC) can be a reliable tool to evaluate PD-L1 expression in tumor samples; however, the use of various clones of PD-L1 and lack of consistent cut-off values complicates comparison among studies for this biomarker [61, 64, 67]. Tumor heterogeneity within the tumor and variation in time and after treatment must also be taken into account. For example, chemotherapy and radiation therapy were shown to affect PD-L1 expression in esophageal cancer [68]. PD-L1 expression increased in a subset of non-small-cell lung carcinoma patients that developed resistance to gefitinib treatment [69]. Lenalidomide, a drug used in the treatment of MM, has been shown to affect the PD-1/PD-L1 axis by downregulating the expression of PD-L1 on MM cells [9, 13] and by decreasing the levels of regulatory T cells and T-cell expression of PD-1 [13, 70, 71]. Proteasome inhibitors bortezomib, carfilzomib, and ixazomib have been shown to increase PD-L1 and PD-L2 levels in MM [34]. The timing of the biopsy is therefore an important consideration.

These factors also complicate the assessment of the value of PD-L1 IHC for predicting response to immune checkpoint blockade. While the expression of PD-L1 by IHC is generally associated with a higher proportion of objective response, [72] patients without expression of PD-L1 can also respond to treatment. The negative predictive value for anti-PD-1 and anti-PD-L1 therapies has been reported to be as low as 58% for nivolumab and 45% for nivolumab plus ipilimumab in melanoma patients [73]. New biomarkers that could better predict response to immune checkpoint blockade include assessment of tumor-infiltrating lymphocytes; T-cell receptor clonality; mutational burden; neoantigen burden; immune gene signatures; multiplex IHC for tumor cells and immune cells, including spatial relationships [73]. Preliminary results of a phase II trial of pembrolizumab monotherapy as consolidation in MM patients (NCT02636010) had demonstrated an upgrade in response in 21% (3/14 patients). Two other patients had improved, but could not yet be considered as having an upgrade in response category. By grouping patients in responders (*n* = 5), progressed (*n* = 5), and stable (*n* = 4), the authors showed that early progression was associated with lower basal NK cells numbers and a lower PD-1 expression in effector memory CD8 cells [74].

A clinical study combining Pembrolizumab, Pomalidomide, and low-dose dexamethasone in relapsed/refractory MM examined PD-L1 expression in bone marrow biopsy of 29 patients by IHC, using a double staining with MUM1 to facilitate identification of plasma cells. There was no correlation with outcome; however, this is a small sample with a median follow-up of 15.6 months. To our knowledge, this was the first study to evaluate PD-L1 in bone marrow using IHC [75]. The cut-off used in this study appear to be the same as those used in lung cancer, i.e., PD-L1 expression in < 1% of the cells considered negative, 1 to 49% weakly positive, and > 50% positive; however, it is unclear if a specific validation of relevant cut-off points in MM was made. Further studies are needed to validate the prognostic and predictive value of IHC for PD-L1. There are also unique challenges in optimizing IHC in bone marrow due to degradation of the antigen during decalcification [76].

### Implications for the design of treatment regimen

PD-L1 impacts prognosis not only through inhibition of immune response, but also by generating intrinsic aggressive characteristics in MM cells [11]. As described earlier, there is evidence that PD-1/PD-L1 blockade alone might not be sufficient in MM and should be combined to other therapeutic strategies. Pre-clinical studies have shown potential benefit of combining PD-1/PD-L1 blockade and immunomodulatory drugs, such as lenalidomide [9, 13]. Lenalidomide has been shown to affect the PD-1/PD-L1 axis by downregulating the expression of PD-L1 on MM cells [9, 13] and by decreasing the levels of regulatory T cells and T-cell expression of PD-1 [13, 70, 71]. However, proteasome inhibitors bortezomib, carfilzomib, and ixazomib have been shown to increase PD-L1 and PD-L2 levels in MM and compromise the anti-myeloma effect of CD8^+^ T cells [34]. Both lenalidomide and proteasome inhibitors are currently used in treatment regimen in clinical practice. Combinations with other immune strategies that stimulate myeloma-reactive T-cell populations, such as tumor vaccines and transplantation [10], or lymphodepleting radiation [77], have also been investigated. Clinical trials involving combinations of chemotherapy regimen and immune checkpoint inhibitors in MM are currently ongoing (Table 2).

**Table 2:** Current clinical trials of PD-1 and PD-L1 inhibitors in multiple myeloma

Treatment	Population	Phase	Status (clinical trial identifier)
Inhibitors of PD-1			
Nivolumab			
Nivolumab + daratumumab with or without pomalidomide + dexamethasone	Hematological malignancies, including MM	Phase 1	Active, not recruiting (NCT01592370)
Nivolumab and ASCT	MM	Phase 1/2	Recruiting (NCT03292263)
Nivolumab + ipilimumab	Treatment-naïve high-risk MM, recurrent MM	Phase 1/2	Recruiting (NCT02681302)
Nivolumab + elotuzumab with or without pomalidomide + dexamethasone	RRMM	Phase 2	Not yet recruiting (NCT03227432)
Elotuzumab + nivolumab versus Elotuzumab + pomalidomide + dexamethasone	RRMM to prior lenalidomide treatment	Phase 2	Active, not recruiting (NCT02612779)
Nivolumab + pomalidomide + dexamethasone versus Nivolumab + elotuzumab + pomalidomide + dexamethasone versus Pomalidomide + dexamethasone	RRMM	Phase 3	Active, not recruiting (NCT02726581)
Pembrolizumab			
Pembrolizumab + lenalidomide + dexamethasone Pembrolizumab + carfilzomib + dexamethasone	Refractory or relapsed and refractory MM	Phase 1 multicohort	Recruiting (KEYNOTE-023/NCT02036502)
Pembrolizumab + lenalidomide	Hematological malignancies, including RRMM	Phase 1	Active, not recruiting (KEYNOTE-013/NCT01953692)
Pembrolizumab + pomalidomide + dexamethasone	RRMM	Phase 1/2	Active, not recruiting (NCT02289222)
Pembrolizumab	MM patients with residual disease	Phase 2	Recruiting (NCT02636010)
Pembrolizumab + ASCT + lenalidomide	MM of any stage with suboptimal response to treatment, prior to transplant	Phase 2	Active, not recruiting (NCT02331368)
Pembrolizumab + lenalidomide + dexamethasone	High-risk MM post ASCT	Phase 2	Active, not recruiting (NCT02906332)
Pembrolizumab + daratumumab	RRMM	Phase 2	Not yet recruiting (KEYNOTE-668/NCT03221634)
Pidilizumab			
Pidilizumab + lenalidomide	RRMM	Phase 1/2	Active, not recruiting (NCT02077959)
Pidilizumab alone or dendritic cell fusion vaccine + pidilizumab after ASCT	MM patients candidate for autologous stem cell transplant	Phase 2	Active, not recruiting (NCT01067287)
PDR001			
CJM112 (anti-IL-17A) alone or with PDR001 PDR001 + LCL161 (oral small molecule SMAC-mimetic)	RRMM	Phase 1	Recruiting (NCT03111992)
JNJ-63723283			
Daratumumab alone or combined with JNJ-63723283	RRMM	Phase 1	Recruiting (NCT03357952)
Inhibitors of PD-L1			
PD-L1 vaccine			
PD-L1 peptide vaccine	MM patient post high-dose chemotherapy with stem cell support	Phase 1	Recruiting (NCT03042793)
Atezolizumab			
Atezolizumab	High-risk asymptomatic myeloma	Phase 1	Recruiting (NCT02784483)
Durvalumab			
Durvalumab alone or combined with Pomalidomide +/− dexamethasone	RRMM	Phase 1	Active, not recruiting (NCT02616640)
Tremelimumab + durvalumab + ASCT	MM at high risk of relapse	Phase 1	Active, not recruiting (NCT02716805)
Durvalumab alone or combined with PVX-410 cancer vaccine with or without lenalidomide	Smoldering MM	Phase 1	Active, not recruiting (NCT02886065)
Durvalumab + daratumumab	RRMM that progressed on daratumumab	Phase 2	Active, not recruiting (NCT03000452)
Durvalumab + daratumumab with or without pomalidomide + dexamethasone	RRMM	Phase 2	Active, not recruiting (NCT02807454)
Suspended trials			
Nivolumab + lenalidomide + dexamethasone	High-risk smoldering MM	Phase 2	Suspended (NCT02903381)
Durvalumab + lenamidomide with or without dexamethasone	Newly diagnosed MM	Phase 1b	Suspended (NCT02685826)
Atezolizumab alone or combined with immunomodulatory drug and/or daratumumab	RRMM and post-ASCT	Phase 1b	Suspended (NCT02431208)
Pomalidomide + dexamethasone with or without pembrolizumab	RRMM	Phase 3	Suspended (KEYNOTE-183/NCT02576977)
Pembrolizumab + lenalidomide + dexamethasone versus lenalidomide + dexamethasone	Newly diagnosed MM	Phase 3	Suspended(KEYNOTE-185/NCT02579863)

A recent review by Jelinek et al. highlighted the various clinical trials that are ongoing for PD-1/PD-L1 inhibitors in hematological malignancies including MM [78]. Published results from a phase 1/2 clinical trial of pembrolizumab, pomalidomide, and low-dose dexamethasone in RRMM (NCT02289222) demonstrated acceptable safety, with objective responses in 60% (29/48) of patients, including stringent complete response/complete response in 8%, with a median duration of response of 14.7 months. The study included 27 high-risk cytogenetically defined patients who had an overall response rate of 56%, with a median progression-free survival of 15.1 months [75]. A number of abstracts have also reported preliminary results from other clinical trials. A phase 1 trial of pembrolizumab, in combination with lenalidomide and low-dose dexamethasone in RRMM (NCT02036502/KEYNOTE-023), showed a tolerable safety profile and 76% (13/17) responded to treatment at a median follow-up of 9.7 months [79]. Pembrolizumab was tested in combination with lenalidomide and dexamethasone in a phase 2 trial of high-risk MM patients, defined by ISS stage 3 and/or high-risk cytogenetic abnormalities and/or high-risk gene expression profile score, 3-6 months post-ASCT. Overall response rate was 100%, and none of the 12 patients had progressed at a median follow-up of 8.5 months [80]. A phase 2 trial evaluated pembrolizumab in 29 patients post-ASCT who had not achieved complete response pre-transplant. The complete response rate was 31% (7 out 23 evaluable patients) at 6 months. The minimal residual disease (MRD)-negative rates by flow cytometry were 44% at day 100 and 67% at end-of-treatment. Post-transplant lenalidomide maintenance therapy was also given as a standard of care [81]. A pilot study of pembrolizumab given to 12 patients with intermediate and high-risk smoldering MM patients, with a median follow-up of 8.54 months, resulted in 1 patient with stringent complete remission, 10 with stable disease and one progression. The patient with stringent complete response had deletion of 17p and amplification of CKS1B, was high-risk by GEP70, and had 50% plasma cell infiltration in the bone marrow at study entry. The authors concluded that immunoprevention with pembrolizumab is tolerable and could possibly prevent progression to MM [82]. A phase 1 trial of nivolumab combined with ipilimumab as consolidation post-ASCT (NCT02681302/CPIT001) demonstrated good response in MM after a median follow-up of 6 months. There were seven transplant-naïve high-risk MM patients, with five stringent complete response, MRD negative; one very good partial response, one progressive disease with death from disease progression. Four MM patients who had relapsed after their first ASCT obtained stringent complete response. The toxicities observed were within expectations for this drug combination, and the immune-related adverse events could be managed with systemic steroids, with only one exception [83]. Interim results from 12 RRMM patients in a phase 1/2 study of pidilizumab combined with lenalidomide (NCT02077959) demonstrated acceptable safety, with 3 very good partial response, 1 partial response, 2 minimal response, and 2 stable disease. Seven patients had been taken off therapy, 6 of them due to disease progression [84].

There were however recent developments that might affect the future of trials involving PD-1 and PD-L1 inhibitors. In June 2017, Merck and the Food and Drug Administration (FDA) suspended enrolment for randomized trials of pembrolizumab with pomalidomide and lenalidomide in relapsed and newly diagnosed patients, based on an imbalance of deaths in the pembrolizumab arms. Two trials (KEYNOTE-183/NCT02576977 and KEYNOTE-185/NCT02579863) were put on full clinical hold due to risks that outweighed any potential benefit for patients [75, 85]. Interim analysis from KEYNOTE-183 (pembrolizumab combined with pomalidomide and low-dose dexamethasone) showed a hazard ratio of 1.61 (95% CI: 0.91, 2.85) for the pembrolizumab-containing investigational arm compared to the control arm, which resulted in over 50% increase in relative risk of death. There was an 18% increase in severe grade 3–5 toxicity, and the incidence of serious adverse events was 63% compared to 46% in the control arm [86]. Interim analysis from KEYNOTE-185 (pembrolizumab combined with lenalidomide and low-dose dexamethasone) showed a hazard ratio of 2.06 (95% CI 0.93, 4.55) for the pembrolizumab-containing investigational arm compared to the control arm, thereby more than doubling the relative risk of death. There was a 22% increase in severe grade 3–5 toxicity, and the incidence of serious adverse events was 54% compared to 39% in the control arm [86].

Trials combining immunomodulators with anti-PD-L1 inhibitors durvalumab (NCT02685826) and atezolizumab (NCT02431208), as well as PD-1 inhibitor nivolumab (NCT02903381), were then suspended based on the safety concerns raised from pembrolizumab trials [87]. Several trials testing drug combinations of immunomodulators with nivolumab, pembrolizumab, or durvalumab in MM were put on partial clinical hold pending further investigation. According to a press release from December 2017, the partial hold was lifted from two trials of nivolumab-based treatment regimen in relapsed/refractory MM (RRMM) [88]. Trials of PD-1/PD-L1 inhibitors with alternative drug combinations are still ongoing; therefore, there might still be a place for PD-1/PD-L1 inhibition in MM. However, these safety concerns provide an incentive to explore alternative treatment combinations and strategies to exploit this pathway in MM.

### New therapeutic strategies targeting PD-L1 expression

Considering the concerns regarding the combination of PD-1/PD-L1 inhibitors with immunomodulators, new strategies are needed to target this pathway. Several molecules have been shown to modulate PD-L1 expression on tumor cells, which can be exploited as monotherapies, combined to other drugs to help potentiate their action or combined to PD-1/PD-L1 inhibitors. The following sections will focus on selected molecules that have been investigated in MM and have shown promising results (Table 3).

**Table 3:** Drugs that modulate the expression of PD-L1 at tumor cell surface

Molecule	Mode of action	Trials in multiple myeloma	Refs.
Lenalidomide	Downregulates PD-L1	Currently used in clinical practice for the treatment of MM Clinical trials: see Table 2 for trials combining lenalidomide to PD-1/PD-L1 inhibitors	[78]
Proteasome inhibitors	Upregulate PD-L1	Currently used in clinical practice Clinical trials: see Table 2 for trials combining lenalidomide to PD-1/PD-L1 inhibitors	[34]
Oncolytic reovirus (Reolysin)	Upregulate PD-L1	Combined with lenalidomide or pomalidomide: phase 1 trial (NCT03015922) Combined with bortezomib and dexamethasone: phase 1b trial (NCT02514382) Combined with PD-L1 blockade: pre-clinical	[17, 90, 91]
HDAC inhibitors	Upregulate or downregulate PD-L1	Pan-HDAC inhibitor panobinostat: approved for RRMM HDAC6 inhibitor combined with lenalidomide and dexamethasone: phase 1b (NCT0158328) HDAC6 inhibitor combined with pomalidomide and dexamethasone: phase 1b (NCT02400242) HDAC6 inhibitor and anti-PD-L1 antibody: pre-clinical HDAC6 inhibitor with anti-PD-1 and lenalidomide: pre-clinical Pan-HDAC inhibitor combined with Reolysin: pre-clinical	[23, 93, 95, 136]
MEK1/2 inhibitor	Blocks the expression of PD-L1 induced by IFN-γ; U0126 can abrogate APRIL-induced expression of PD-L1 on MM cells	Pre-clinical (U0126) Trametinib with dabrafenib: phase 1 (NCT03091257) Trametinib with AKT inhibitor GSK2110183: phase 1 (NCT01476137) Selumetinib: phase 2 (NCT01085214) Binimetinib with encorafenib: phase 2 (NCT02834364) Trametinib with GSK2141795: phase 2 (NCT01989598)	[14, 35, 99, 100]
Anti-APRIL monoclonal antibody hAPRIL01A	Downregulates PD-L1	Pre-clinical	[35, 97]
APRIL CAR T cells	Target BCMA and TACI	Phase 1/2 (NCT03287804)	
Anti-BCMA	Downregulates PD-L1 (APRIL/BCMA signaling cascade)	Pre-clinical (GSK2857916) GSK2857916: phase 1 (NCT02064387) SEA-BCMA combined with ACTR087: phase 1 (NCT03266692) JNJ-64007957: phase 1(NCT03145181) PF-06863135: phase 1 (NCT03269136)	[96]
BCMA CAR T cells	Target BCMA	LCAR-B38M: clinical trial bb2121: phase 1 (NCT02658929) CART-BCMA: phase 1 CART- BCMA: phase 1 (NCT02215967)	[102–105]
BET inhibitors	Downregulate PD-L1	OTX015: phase 1 (NCT01713582) Ongoing phase 1 clinical trials (Pre-clinical in pancreatic and ovarian cancer, mouse model of lymphoma)	[106–109]
STAT3/BTK inhibition	Downregulates PD-L1	Ibrutinib and anti-PD-L1: pre-clinical BBI608: phase 1 (NCT02352558) Ibrutinib with carfilzomib: phase 1/2 (NCT01962792) Ibrutinib, lenalidomide, and dexamethasone: phase 1/2 (NCT03015792) Ibrutinib, pomalidomide, and dexamethasone: phase 1/2 (NCT02548962) Ibrutinib, bortezomib, and dexamethasone: phase 2 (NCT02902965) Ibrutinib (high-risk smoldering MM): phase 2 (NCT02943473) (WP1066: pre-clinical in lymphoma; Ibrutinib: pre-clinical in chronic lymphocytic leukemia)	[110–112]
MUC1-C inhibitors	Increase miR-34a and miR-200c, causing decreased expression of PD-L1	Tecemotide vaccine: phase 2 (NCT01094548) ImMucin vaccine with hGM-CSF: phase 1/2 (NCT01232712) (GO-203: pre-clinical in acute myeloid leukemia model, pre-clinical in lung cancer model)	[122, 123, 125]

#### Oncolytic reovirus

Oncolytic virus therapy is a therapeutic strategy that has gained interest in the treatment of MM [89]. Reolysin is an oncolytic reovirus-based anticancer agent that has shown acceptable safety in MM patients in a phase 1 trial [90]. There is an ongoing phase 1 trial of Reolysin in combination with lenalidomide or pomalidomide (NCT03015922), as well as a phase 1 trial of Reolysin with carfilzomib and dexamethasone (NCT02101944). There is also an ongoing phase 1b trial of Reolysin combined with Bortezomib and dexamethasone in RRMM (NCT02514382). Preliminary results for six patients showed stable disease in three patients and progressive disease in three patients [91]. Kelly et al. showed that Reolysin triggers a significant transient upregulation of PD-L1 in MM cell lines and in vivo, which could have mitigated treatment response in the clinical trial. They hypothesized that this drug could be used as a priming strategy to potentiate PD-1/PD-L1 therapy. The combination of Reolysin and anti-PD-L1 therapy produced a significant decrease in disease burden and increased overall survival in a mouse model. These results provide a rationale for combining Reolysin and PD-1/PD-L1 inhibitors in clinical trials [17].

#### Histone deacetylase inhibitors

Histone deacetylase (HDAC) inhibitors are a new class of drugs that has shown promising results in the treatment of MM. There are 70 clinical trials involving HDAC inhibitors in MM listed on clinicaltrials.gov as of 2 January 2018 [87]. Interestingly, HDAC inhibitors have been shown to upregulate or downregulate the expression of PD-L1 and PD-L2 in multiple cancer cell lines, the effects seemingly variable depending on the class and isotype of HDAC that is being targeted. HDAC inhibitors were also shown to enhance the efficacy of anti-PD-1 antibodies in an animal model of melanoma [24, 92]. The combination of HDAC6 and PD-1 inhibitors is currently under trial in melanoma (NCT02935790). More recently, the combination of HDAC6 inhibitor and anti-PD-L1 antibody was shown to trigger cytotoxic T lymphocytes and NK cells mediated MM cell killing [23]. A combination of HDAC6 inhibitor, anti-PD-1 and lenalidomide further enhanced HDAC6 inhibition-induced effector cell-mediated anti-MM immune response in MM cells [93]. It has also been shown that HDAC3 silencing in bone marrow stromal cells (BMSC) is associated with a significant decrease in BMSC-induced MM proliferation. This effect was reproduced with MM cells cultured in conditioned media from HDAC3-silenced BMSC, suggesting a paracrine effect. The supernatant also inhibited the proliferation of activated T-cells. Preliminary data showed a 1.37-fold increase in soluble PD-L1 in the supernatant when HDAC3 was silenced in HS-5 stromal cells. These results provide a rational for combining immune checkpoint inhibition and HDAC3 selective inhibitors, such as BG45 [94].

A study also showed that pan HDAC inhibitors sensitize MM cells to Reolysin in vitro and in vivo by increasing the expression of reovirus receptor JAM-1. This combination increased reovirus replication in MM cells and was associated with significantly decreased bone marrow infiltration in a mice model [95]. Since some HDAC inhibitors have been shown to downregulate PD-L1, perhaps they also mitigate the increased PD-L1 expression described with Reolysin treatment.

#### Inhibition of APRIL/BCMA/MEK/ERK pathway

BCMA is a selective antigen found on plasma cells and plasmacytoid dendritic cells, making it an interesting target for the treatment of MM [96]. It has been shown to promote growth and survival of MM cells [97]. APRIL, a ligand of BCMA, has been shown to upregulate PD-L1 in MM cells via a MEK/ERK pathway [35]. An anti-APRIL monoclonal antibody, hAPRIL01A, could abolish MM cell growth and drug resistance, as well as prevent MM development in a mouse model. It could also trigger cytotoxicity against MM cells and enhance the activity of lenalidomide or bortezomib [97].

Conversely, MEK1/2 inhibitor U0126 was able to block APRIL-induced PD-L1 expression in MM cell lines [35]. U0126 could also block the expression of PD-L1 induced by IFN-γ in MM plasma cells and restore T-cell function [14]. Similar findings were observed in non-small cell lung carcinoma cell lines [98]. Selumetinib, a MEK1/2 inhibitor, showed minimal activity in RRMM patients in a phase 2 study (NCT01085214) [99]. There are ongoing phase 2 trials of MEK inhibitor binimetinib combined with kinase inhibitor encorafenib in RRMM with BRAFV600E/K mutation (NCT02834364), and MEK1/2 inhibitor trametinib with Akt inhibitor GSK2141795 in RRMM (NCT01989598). There is also a phase 1 trial of trametinib and/or RAF kinase inhibitor dabrafenib in RRMM (NCT03091257). Preliminary results from phase 1 trial of trametinib with AKT inhibitor GSK2110183 (NCT01476137) revealed issues with tolerability, but there seems to be only one patient with MM in this cohort [100].

Because of its relatively selective expression in plasma cells, BCMA has gained interest as a target for chimeric antigen receptor (CAR) T cell therapy. CAR T cells are synthetically engineered T cells that express artificial CARs that target a tumor cell surface molecule. They can therefore recognize tumor cells even if they do not express human leucocyte antigen molecules [101]. There are 18 ongoing trials of BCMA CAR T cells in MM listed on clinicaltrials.gov [87]. Results from a dose escalation trial of CAR-BCMA T cells in MM with uniform BCMA expression by IHC or flow cytometry demonstrated antimyeloma activity, with complete remission seen in one patient for 17 weeks before relapse and one patient with very good partial remission that lasted 66 weeks [102]. Preliminary results from three other trials are available. Results from six patients in a phase 1 trial of CART-BCMA cells showed one complete remission, one minimal response of 2 months, one minimal response, and no response in two patients. One patient had progression with loss of BCMA expression on MM cells, suggesting antigen escape [103]. A trial of LCAR-B38M CAR T cells targeting BCMA in MM showed 100% objective response rate in 19 RRMM patients, with 18 having complete or near complete remission at a median follow-up of 6 months [104]. A multi-center phase 1 trial of bb2121, a CAR T cell construct targeting BCMA, in RRMM patients showed 100% overall response rate in six evaluable patients (NCT02658929) [105]. There is one phase 1/2 clinical trial of APRIL CAR T cells in RRMM currently ongoing (NCT03287804).

#### Bromodomain and extraterminal inhibitors

Pancreatic tumor cells treated with mimics of miR-93-5p and miR-106b-5p showed reduced expression of PD-L1. This effect could be reproduced by treatment with bromodomain and extraterminal (BET) inhibitor OTX015 [106]. Downregulation of PD-L1 by a panel of BET inhibitors was also shown in ovarian cell lines and a mouse model of ovarian carcinoma in a dose- and time-dependent manner [107]. This was further confirmed in a study that showed that BET inhibitors trigger suppression of constitutively expressed or IFN-γ induced PD-L1. BRD4, a molecule associated with active promoters and enhancers, was a critical regulator of PD-L1 expression, independent of MYC. Of note, this effect was not found in all cell types, such as mouse MLL-rearranged AML and in some Eμ-Myc lymphomas. There was a synergistic effect of BET inhibitor JQ1 and anti-PD-1 antibody in a mouse model of Eμ-Myc lymphoma, providing evidence for the relevance of combining BET inhibitors and immune checkpoint inhibitors [108]. A phase 1 open-label study of OTX015 in patients with hematological malignancies showed no activity in the 12 MM patients, 2 of them having stable disease and the other 10 having progression as best response (NCT01713582). It was pointed out that this is a small sample and half were treated at suboptimal doses [109]. There are five other phase 1 clinical trials of BET inhibitors involving MM patients that are currently ongoing as listed on the site clinicaltrials.org [87].

#### STAT3 and Bruton tyrosine kinase inhibition

A STAT3 inhibitor, WP1066, could abrogate PD-L1 expression in lymphoma cell lines, leading to decreased cell growth and increased cell apoptosis [110]. There is an ongoing clinical trial of STAT3 inhibitor BBI608 alone or with bortezomib or dexamethasone in RRMM (NCT02352558).

Ibrutinib, a covalent inhibitor of Bruton tyrosine kinase (BTK), caused selective and durable downregulation of PD-L1 on chronic lymphocytic leukemia cells within 3 months of therapy, which was mediated through the inhibition of constitutively active STAT3 on these cells [111]. The combination of Ibrutinib and an antibody against PD-L1 in an animal model of lymphoma with no intrinsic sensitivity to Ibrutinib resulted in cure of half the mice and prolonged survival in the remaining ones. These results were also confirmed in the J558 mouse model of plasma cell neoplasm. They did not observe any alteration of PD-L1 surface expression on cell lines following administration of Ibrutinib for 12 h and in tumor cells extracted form mice 1 h after treatment [112]. Perhaps the duration of treatment can explain the discrepancy with the study in chronic lymphocytic leukemia. There are five ongoing phase 1/2 and phase 2 clinical trials of Ibrutinib alone or in combination in RRMM, MM ineligible for transplant and high-risk smoldering MM (NCT01962792, NCT03015792, NCT01478581, NCT02548962, NCT02902965, NCT02943473) [87].

#### MicroRNAs

MiRNAs represent another class of molecules that have gathered interest as a target for cancer therapy. A comprehensive review of miRNAs involved in PD-L1 regulation is outside the scope of this paper; however, there have been two recent reviews on the topic by Wang et al. and Smolle et al. [18, 113]. Some of the most significant miRNAs regulating PD-L1 expression are the miR-34a, miR-200, and miR-197. Some other miRNAs such as the miR15 family, miR-20b, miR-21, miR-93, miR-106b, miR-130b, miR-138-5p, miR-142-5p, miR-152, miR-193a-3p, miR-324-5p, miR-338-5p, miR-513, and miR-570 can also regulate PD-L1 expression in different cancer types [18, 113]. More recently, miR-17-5p was shown to post-transcriptionally regulate PD-L1 in melanoma [58]. A pathway involving IFN-γ/TNF-α/miR-155/PD-L1 was demonstrated using dermal lymphatic endothelial cells [114]. Another pathway involving miR-217/AEG-1/PD-L1 was found to inhibit metastatic traits in laryngeal cancer cells [115]. MiR-195 was found to regulate PD-L1 in diffuse large B-cell lymphoma [116]. There is also a number of miRNAs that target various regulators of PD-L1 expression, such as IFN-γ, PTEN, mTOR, or STAT1 [18]. The miR-197/CKS1B/STAT3-mediated PD-L1 network has been identified in chemoresistant non-small cell lung carcinoma. A mimic of miR-197 could sensitize PD-L1^high^ drug-resistant cells to chemotherapy, thereby suggesting a role for miRNA replacement therapy [117].

To our knowledge, miRNAs involved in regulation of PD-L1 in MM have yet to be described; however, considering the overlap with some miRNAs known to be involved in MM, such as the miR-15 family, the miR17~ 92 cluster, miR-21, miR-34a, or miR-106b [118, 119], it stands to reason that some miRNAs regulating PD-L1 expression could be involved in MM. MiR-34 therapy has been shown to result in increased survival in vitro and in MM animal models [120, 121]. It was also found to enhance the anti-tumor activity of γ-secretase inhibitor and HDAC inhibitor Sitrinol in MM cells through pro-apoptotic effects [119]. The expression of PD-L1 was not assessed in these studies; however, considering that miR-34a is a known regulator of PD-L1 expression and that PD-L1 expression can impact apoptosis, it would be interesting to investigate if the anti-MM effects of miR-34a can be partly attributed to the modulation of PD-L1 expression.

Notably, treatment of acute myeloid leukemia cells with MUC1-C inhibitor peptide (GO-203) caused an increase in miR-34a and miR-200c, which lead to a decreased expression of PD-L1 and decreased leukemia involvement in an animal model [122]. G0-203 was also found to downregulate PD-L1 expression in non-small cell lung carcinoma cells [123]. MUC1, a mucin molecule, is expressed in about 50% of MM [124]. A clinical trial investigated tecemotide, a vaccine targeting MUC-1, in 34 patients with slowly progressive asymptomatic MM that never received chemotherapy (NCT01094548). It was well tolerated and induced MUC-1-specific cellular immune response in about 50% of patients; however, this response was weak and of poor durability. There was no objective clinical response, but preliminary evidence was consistent with disease stabilization in a subset of patients without a pre-existing MUC1-specific immune response. The results of this exploratory study would need to be confirmed in a larger cohort, perhaps in combination with immunomodulatory therapy [125]. Another MUC1 peptide vaccine, ImMucin, is being studied in combination with recombinant human granulocyte-monocyte colony stimulating factor (hGM-CSF) in MM patients with MUC1 expression (NCT01232712).

## Conclusions

Interesting results have been obtained in clinical trials combining PD-1 inhibitors with immunomodulators in MM; however, concerns regarding the safety of these combinations have been raised and several clinical trials have been suspended or put on hold. There has been increasing interest in new therapeutic strategies directly targeting the expression of PD-L1 on cancer cell surface, which could be a relevant approach in MM. Further studies aiming to target the molecules involved in the regulation of PD-L1 in MM will allow the design of new drugs or the application of available drugs that can affect the expression of PD-L1. While PD-L1 expression is relatively restricted in normal tissue, targeting downstream molecules involved in its regulation could potentially be associated with more widespread adverse effects. This will need to be addressed in ongoing and future clinical trials. However, promising results have been obtained in clinical trials with CAR T cells targeting BCMA, HDAC inhibitors, and oncolytic reovirus, supporting the relevance of these alternative strategies in the treatment of MM. As research on the PD-1/PD-L1 axis continues, a variety of molecules are found to have a role in the regulation of PD-L1 expression, such as Sigma1 inhibitor IPAG [126], serine protease inhibitor nafamostat mesilate [127] or VEGFR-2 inhibitor Apatinib [128], and there is a rationale for investigating their application in MM. Drugs modulating the expression of PD-L1 could represent new therapeutic avenues for the treatment of MM, help potentiate the action of other drugs or be combined to PD-1/PD-L1 inhibitors in order to avoid the potentially problematic combination with immunomodulators.

## Abbreviations

GSK3β: Glycogen synthase kinase 3β
IFN-γ: Interferon-γ
APRIL: A proliferation-inducing ligand
ASCT: Autologous stem cell transplant
BCMA: B-cell maturation antigen
BET: Bromodomain and extraterminal
BMSC: Bone marrow stem cell
BTK: Bruton tyrosine kinase
CAR: Chimeric antigen receptor
CSN5: COP9 signalosome 5
EGF: Epidermal growth factor
hGM-CSF: Human granulocyte-monocyte colony stimulating factor
IHC: Immunohistochemistry
MGUS: Monoclonal gammopathy of undetermined significance
miRNA: MicroRNAs
MM: Multiple myeloma
MRD: Minimal residual disease
NK: Natural killer
PD-1: Programmed cell death-1
PD-L1: Programmed death-ligand 1
RRMM: Relapsed/refractory multiple myeloma
